# A Novel Controlled Fabrication of Hexagonal Boron Nitride Incorporated Composite Granules Using the Electrostatic Integrated Granulation Method

**DOI:** 10.3390/nano13010199

**Published:** 2023-01-02

**Authors:** Taisei Nakazono, Atsushi Yokoi, Wai Kian Tan, Go Kawamura, Atsunori Matsuda, Hiroyuki Muto

**Affiliations:** 1 Department of Electrical and Electronics Information Engineering, Toyohashi University of Technology, Toyohashi 441-8580, Aichi, Japan; 2 Institute of Liberal Arts and Sciences, Toyohashi University of Technology, Toyohashi 441-8580, Aichi, Japan

**Keywords:** electrostatic nano-assembly, granulation, composite, heat-conduction, hexagonal boron nitride, alumina

## Abstract

Despite the availability of nano and submicron-sized additive materials, the controlled incorporation and utilization of these additives remain challenging due to their difficult handling ability and agglomeration-prone properties. The formation of composite granules exhibiting unique microstructure with desired additives distribution and good handling ability has been reported using the electrostatic integrated granulation method. This study demonstrates the feasible controlled incorporation of two-dimensional hexagonal boron nitride (hBN) sheets with alumina (Al_2_O_3_) particles, forming Al_2_O_3_–hBN core–shell composite granules. The sintered artifacts obtained using Al_2_O_3_–hBN core–shell composite granules exhibited an approximately 28% higher thermal conductivity than those obtained using homogeneously hBN-incorporated Al_2_O_3_ composite granules. The findings from this study would be beneficial for developing microstructurally controlled composite granules with the potential for scalable fabrication via powder-metallurgy inspired methods.

## 1. Introduction

With technological advancement, device miniaturization and dense electronic component packaging have led to heat dissipation problems in electronic devices. Poor heat dissipation leads to overheating, adversely affecting the performance, service life and user safety. With the increased demand for power electronics, developing heat dissipation materials has been given attention. 

The development of heat-conductive and electrically insulative composites has been carried out to improve heat dissipation within electronic devices by incorporating inorganic fillers such as alumina (Al_2_O_3_) [1] and boron nitride (BN) [2,3]. Hexagonal BN (hBN) is a good heat-conductive material as it exhibits excellent thermal conductivity and good electrical insulation. The unique honeycomb-configured sp^2^-bonded boron and nitrogen promote anisotropic thermal conductivity with in-plane and out-of-plane thermal conductivity of 600 and 30 W·m^−1^·K^−1^, respectively [4,5]. Due to the high mechanical strength, chemical stability, and high thermal conductivity of hBN, it is widely used in different applications such as high-temperature ceramics and electronic packaging [6]. 

However, the heat flow vector distribution in hBN is significantly influenced by the orientation of hBN grains or sheets. When the hBN layer’s direction is at a small angle against the direction of the external temperature gradient, the heat flow propagates along the in-plane conductive pathway, while at a different angle direction, hBN acts as a heat insulator [7].

Therefore, controlled hBN distribution within its matrix is important to effectively utilize the high thermal conductivity of hBN for heat conduction within their composites. The electrostatic nano-assembly method has been demonstrated to be useful for designing nano- and microstructured composites exhibiting controlled additive distribution [8,9,10,11]. In recently reported studies, it has been demonstrated that a controlled decoration of submicron and micro-sized hBN on poly(methylmethacrylate) (PMMA) and vice versa, is feasible to obtain a controlled homogeneous microstructure exhibiting improved thermal conductivity [2,3,5]. The aforementioned studies reported the decoration of smaller hBN sheets on larger PMMA particles via formation of composite particles. However, this method is difficult to be used when the particle size of the starting materials for the matrix is smaller than the size of hBN sheets. 

Thus, in this study, using the electrostatic integrated granulation (EIG) method, the formation of composite granules consisting of Al_2_O_3_ particles (average particle size: 140 nm) and hBN sheets (average particle size: 5 µm) is demonstrated. A recent study reported the formation of composite granules consisting of one-dimensional carbon nanotubes (CNT) with two different oxide particle types, demonstrating a controllable microstructure formation using the EIG method. Homogeneous CNT incorporation or only at the shell region of the composite granules was obtained by adjusting the CNT incorporation step, and the final sintered artifacts exhibited a considerable difference in electrical conductivity [12].

No study has reported the controlled incorporation of two-dimensional sheet-like hBN with oxide particles (Al_2_O_3_) in the form of composite granules; thus, it is interesting to investigate the feasibility and the heat-conductive property of sintered artifacts. With the improved handling ability of composite granules and enhanced heat conduction of sintered artifacts, hBN-incorporated composite granules can be useful for fabricating heat-conductive ceramic composites using powder metallurgy-inspired methods that possess a good potential for scalable manufacturing.

## 2. Materials and Methods

Hexagonal boron nitride (hBN) sheets (average particle size of 5 µm, Showa Denko Co., Ltd., Tokyo, Japan) and alumina particles (Al_2_O_3_, average particle size of 140 nm, Taimei Chemical Co., Ltd., Tokyo, Japan) were used in this study. The polycation and polyanion used were polydiallyldimethyl ammonium chloride (PDDA) (average molecular weight 100,000 to 200,000, Sigma-Aldrich, St. Louis, MO, USA) and polysodium styrenesulfonate (PSS) (average molecular weight 70,000, Sigma-Aldrich, St. Louis, MO, USA), respectively. The surfactant used for the initial coating onto hBN was sodium deoxycholate (SDC). 

**Figure 1:**
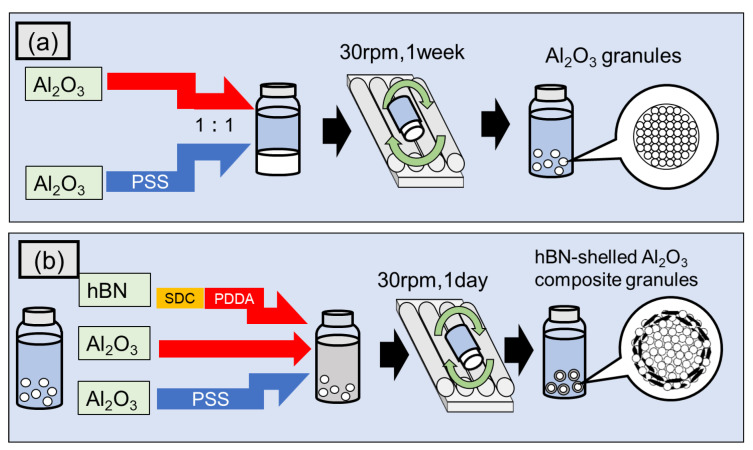
The fabrication schematic of the (**a**) Al_2_O_3_ granules and (**b**) Al_2_O_3_–hBN core–shell composite granules using the electrostatic integrated granulation method.

As Al_2_O_3_ particles exhibit a positive surface charge in aqueous suspensions, a negatively charged Al_2_O_3_ suspension was prepared by adding PSS polyanion. Positively and negatively charged Al_2_O_3_ suspensions with a volume fraction of 1:1 were mixed into a 9 mL glass vessel and rotated at 30 rpm for one week to form composite granules consisting of only Al_2_O_3_, as shown in Figure 1a. The obtained Al_2_O_3_ granules were then used as the core for the subsequent shell layer formation consisting of hBN and Al_2_O_3_ particles to form composite granules with a structure comprising the Al_2_O_3_ core and the hBN–Al_2_O_3_ shell (Al_2_O_3_–hBN CS). The two-dimensional hBN sheets dispersed in an SDC solution using a homogenizer (QSonica, LLC., Q700, Newtown, CT, USA) for 10 min, were put into a PDDA solution to induce a positively charged hBN aqueous suspension. Then, Al_2_O_3_ granules were inserted into a 9 mL glass vessel with an aqueous suspension mixture consisting of positively charged Al_2_O_3_, hBN, and negatively charged Al_2_O_3_ at a volume fraction of 4:1:5. The hBN amount incorporated against Al_2_O_3_ particles was approximately 3.95 vol.%. After that, the glass vessel was rotated at 30 rpm for one day to form the Al_2_O_3_–hBN CS composite granules, as shown in Figure 1b. As for the fabrication of homogeneously incorporated Al_2_O_3_–hBN composites granules (similar hBN amount at 3.95 vol.%), the suspensions of hBN sheets and Al_2_O_3_ particles (with surface charge positively and negatively adjusted) were mixed simultaneously and rotated for one week at 30 rpm. The fabricated composite granules were collected and dried in an oven at 80 °C for 24 h.

For fabrication of sintered artifacts, the composite granules obtained were uniaxially pressed at 40 MPa and sintered at 1250 °C for 10 min using a spark plasma sintering equipment (SS-Alloy Plasma Kit, CSP-KIT-02121, Hiroshima, Japan). The zeta potential was measured using zeta potential measuring equipment (Otsuka Electronics, ELSZ-1, Osaka, Japan). A laser microscope (Olympus, OLS 4100, Tokyo, Japan) and a field-emission scanning electron microscope (FE-SEM, Hitachi S-4800, Tokyo, Japan) were used for morphological observation of the composite granules. The crystal structure of the raw powders and the spark-plasma-sintered artifacts was characterized by X-ray diffraction (XRD) (Ultima IV, Rigaku Co., Ltd., Tokyo, Japan). The thermal conductivity of the sintered artifacts was measured using the laser flash method (Xenon Flash Apparatus Analyzer, Model XFA 300/600 LINSEIS, Vielitzerstr, Germany) [3,13]. 

## 3. Results and Discussion

**Figure 2:**
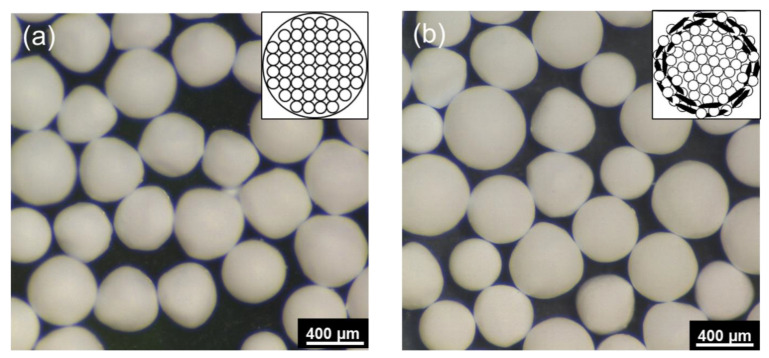
Optical microscope images of the dried (**a**) Al_2_O_3_ granules and (**b**) Al_2_O_3_–hBN CS composites granules. Insets are representative schematics for their internal microstructure.

The optical microscope images of the core Al_2_O_3_ granules and Al_2_O_3_–hBN CS composite granules are shown in Figure 2a,b, respectively. Schematic representation of the granules’ microstructure is also shown as an inset in each respective figure. These images show that almost-spherical Al_2_O_3_ granules and Al_2_O_3_–hBN CS composite granules were obtained using the EIG method. Interestingly, even though two-dimensional hBN sheets were incorporated in the Al_2_O_3_–hBN CS composite granules, spherically structured composite granules were obtained. The average diameter of the granules obtained ranged from 300 to 600 µm.

**Figure 3:**
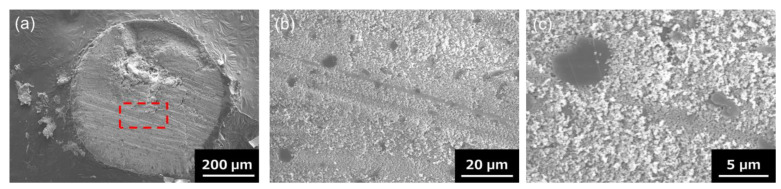
(**a**) Cross-sectional SEM images of a homogeneously hBN-incorporated Al_2_O_3_ composite granule. (**b**) The higher magnification image of the region marked with the red-dotted line in (**a**). (**c**) Zoomed-in SEM image of an area in (**b**) indicating the presence of hBN sheets.

Cross-sectional SEM observations of the composite granules were carried out to confirm the distribution of hBN sheets within homogeneously hBN-incorporated Al_2_O_3_ composite granules and the presence of hBN sheets at only the shell layer of Al_2_O_3_–hBN CS composite granules. A cross-sectional SEM image of a homogeneously hBN-incorporated Al_2_O_3_ composite granule is shown in Figure 3a. hBN sheets can be observed to be uniformly distributed among Al_2_O_3_ particles from the higher magnification images of Figure 3b,c. 

**Figure 4:**
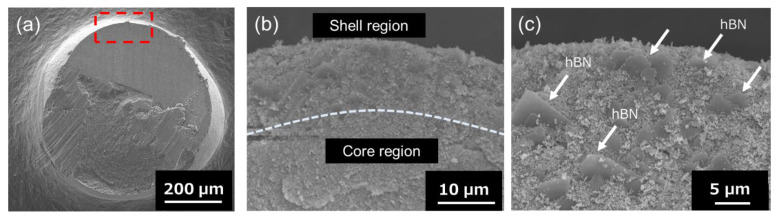
(**a**) Cross-sectional SEM images of an Al_2_O_3_–hBN CS composites granule. (**b**) The higher magnification image of the interface between the core and shell region, marked with the red-dotted line in (**a**). The interface between the core and shell region is marked with dotted line. (**c**) Zoomed-in SEM image of the shell region indicating the presence of hBN sheets (arrow marked).

Figure 4 shows the SEM images of the Al_2_O_3_–hBN CS composite granule at low and high magnifications. The area which is marked (red-dotted line) in Figure 4a is observed at higher magnification and is shown in Figure 4b. From the higher magnification SEM image of Figure 4b, two distinct regions (marked with dotted line) can be observed, which are the core and the shell region. The thickness of the shell region was approximately 6 µm. Further magnified SEM image of the shell region is shown in Figure 4c, which clearly shows the presence of hBN sheets, which were embedded within Al_2_O_3_ particles. At the core of the composite granules, only Al_2_O_3_ particles were observed, and there was no presence of hBN sheets, as shown in Figure S1. This indicates a feasible control of hBN sheet incorporation in Al_2_O_3_–hBN CS composite granules using the EIG method. This result demonstrates a controlled incorporation of two-dimensional microstructured sheets with ceramic particles forming core–shell microstructure composite granules for the first time.

**Figure 5:**
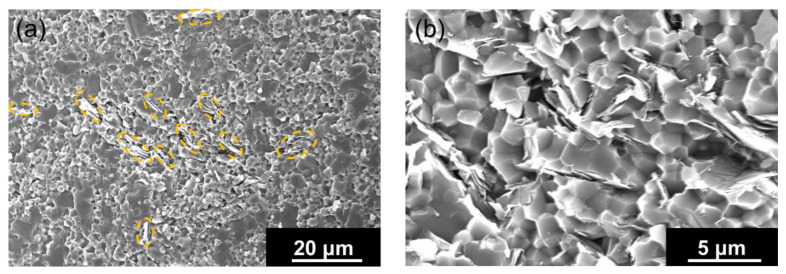
(**a**) The microstructure of the sintered artifact obtained using homogeneously hBN-incorporated Al_2_O_3_ composite granules, indicating the uniform hBN distribution (dotted circles) within the alumina matrix. (**b**) The high-magnification SEM image of an area with hBN sheets.

The microstructure of the sintered artifact obtained using homogeneously hBN-incorporated Al_2_O_3_ composite granules is shown in Figure 5. Figure 5a shows a rather uniform distribution of hBN sheets (marked with dotted circles) within the Al_2_O_3_ matrix. The higher magnification SEM image shown in Figure 5b clearly indicates the presence of hBN sheets. 

**Figure 6:**
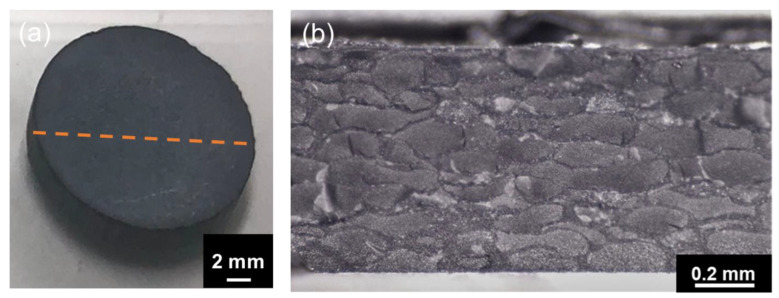
(**a**) The photograph of a sintered artifact obtained using Al_2_O_3_–hBN CS composite granules. (**b**) The cross-sectional optical microscope image taken at the marked line (dotted) in (**a**) showing a unique interconnected mesh-like microstructure.

**Figure 7:**
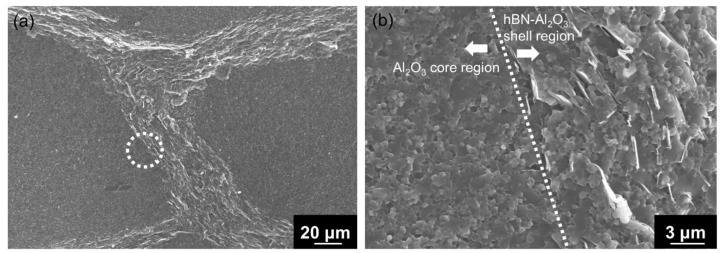
(**a**) The cross-sectional SEM image showing the boundary of a sintered artifact using Al_2_O_3_–hBN CS composite granules. (**b**) The high-magnification SEM image taken at the grain boundary marked in (**a**) with a circle (dotted line), indicating a distinguished interface (straight dotted line) between the Al_2_O_3_ core region (left) and Al_2_O_3_–hBN shell region (right).

The spark-plasma-sintered artifact obtained using Al_2_O_3_–hBN CS composite granules is shown in Figure 6. A rather solid sintered artifact was obtained after sintering as shown in the photograph in Figure 6a. From the cross-sectional view of the sintered Al_2_O_3_–hBN composite pellet observed using an optical microscope, a granular structured microstructure with a mesh-like interconnected layer formed in between can be observed as shown in Figure 6b. A detailed observation of the mesh-like interlayer was carried out using SEM and the images obtained are shown in Figure 7. At a lower magnification SEM image shown in Figure 7a, the presence of interlayer can be clearly observed and a high magnification of the area with a dotted circle, which consists of the core and shell regions, was taken and is shown as Figure 7b. Figure 7b shows a distinct difference between the core Al_2_O_3_ region and the Al_2_O_3_–hBN composite shell region, which is marked with a straight dotted line. From these observations, sintering of Al_2_O_3_ particles was achieved, and higher magnification images are shown in Figure S2, showing the coalescence of Al_2_O_3_ particles. Besides that, it is noteworthy that hBN sheets appeared to exhibit a designated orientation, consistent with the observation of an Al_2_O_3_–hBN CS composite granule in Figure 4c. This phenomenon could be caused by the size segregation developed in the viscous fluid flow during the EIG process [14]. In addition, the crowding of electrostatically integrated agglomerates could cause a flow-induced alignment of the sheets during the granulation process. Calabrese et al. reported on a similar observation in the alignment of colloidal rods suspended in crowded polymer solution, caused by the polymer dynamics generating shear-induced alignment of colloidal rods suspended in the polymer solution [15].

**Figure 8:**
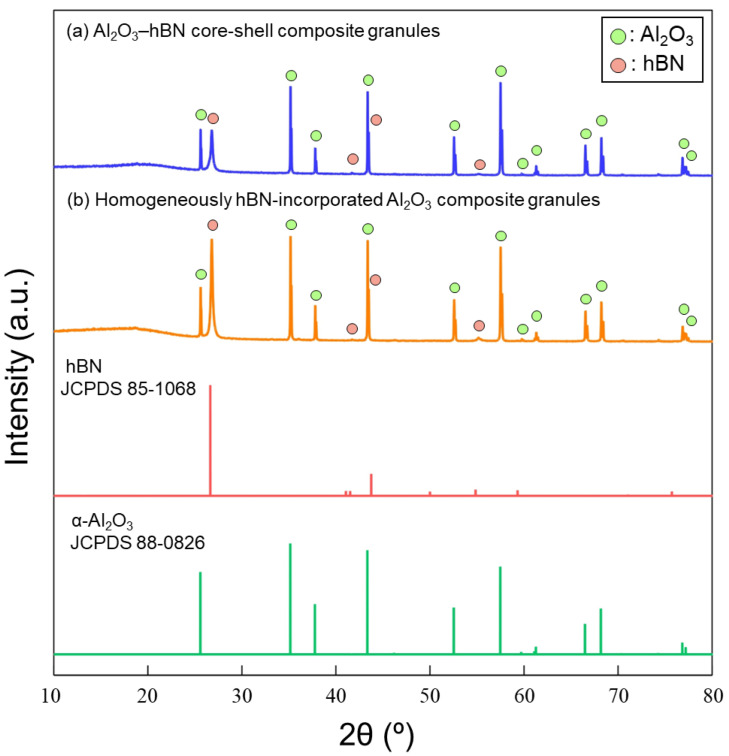
XRD patterns of the sintered composite artifacts obtained using (**a**) Al_2_O_3_–hBN core–shell composite granules and (**b**) homogeneously hBN-incorporated Al_2_O_3_ composite granules.

XRD characterizations were carried out to confirm the crystallinity of the sintered composite artifacts. XRD of the raw materials was also done, and the XRD patterns are shown in Figure S3, corresponding to α-Al_2_O_3_ (JCPDS 88-0826) and hBN (JCPDS 85-1068). The XRD patterns of the sintered artifact obtained using homogeneously hBN-incorporated Al_2_O_3_ composite granules and Al_2_O_3_–hBN CS composite granules are shown in Figure 8. The XRD patterns showed only peaks of the incorporated materials, α-Al_2_O_3_ (JCPDS 88-0826) and hBN (JCPDS 85-1068). This indicates no other phase or impurity was formed after the sintering. XRD patterns of the sintered composite artifacts are consistent with those of the raw powders used. 

The thermal conductivity of the sintered artifacts obtained using the Al_2_O_3_–hBN CS composite granules and homogeneously hBN-incorporated Al_2_O_3_ composite granules was evaluated. The hBN amount incorporated in both composite granules was the same at 3.95 vol.%. Table 1 shows the thermal conductivity of the sintered artifacts obtained using Al_2_O_3_–hBN composite granules. 

From Table 1, the thermal conductivity of homogeneously hBN-incorporated Al_2_O_3_ and Al_2_O_3_–hBN CS sintered artifacts was 11.65 and 14.90 W/mK, respectively. The results showed that by adjusting the hBN sheet distribution within the composite granules during the EIG process, a unique sintered interconnected microstructure was obtained, enabling improved thermal conduction. The thermal conductivity increased from 11.65 to 14.90 W/ mK, marked an approximate 28% increment despite incorporating the same hBN amount (3.95%).

In this study, the formation of composite granules with controlled incorporation of two-dimensional hBN sheets with Al_2_O_3_ particles is demonstrated using the EIG method. With a more controlled distribution of hBN sheets at the shell region, an improved thermal conductivity of sintered composite artifacts was obtained. The efficient utilization of heat-conductive sheet-like additives and a feasible control of thermal conductivity using composite granules could be beneficial for developing heat-conductive composites via a powder metallurgy-inspired route. 

## 4. Conclusions

With the increased demand for the large-scale fabrication of heat-conductive composites, the efficient usage of heat-conductive additives with good handling ability for efficient and scalable processing, such as the powder metallurgy method, is required. In this study, the controlled incorporation of two-dimensional hBN sheets into an Al_2_O_3_ matrix is demonstrated using composite granules obtained by the EIG method. Sintering of the Al_2_O_3_–hBN core–shell composite granules led to the formation of a unique interconnected microstructure, promoting better heat conduction and leading to improved thermal conductivity. A 28% increment in thermal conductivity was obtained using Al_2_O_3_–hBN core–shell composite granules compared with the sintered artifact obtained using homogeneously hBN-incorporated Al_2_O_3_ composite granules. The feasibility of incorporating additives with different dimensions that lead to the creation of desired properties using composite granules could be beneficial for the large-scale manufacturing of advanced composites.

## Figures and Tables

**Table 1:** Comparison of the thermal conductivity of sintered artifacts obtained using two different types of Al_2_O_3_–hBN composites granules.

Type of Granules	Schematic Representation	Volume Fraction of Incorporated hBN Sheets (vol.%)	Thermal Conductivity (W/mK)
Homogeneously hBN-incorporated Al_2_O_3_ composite granules	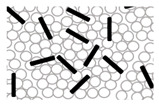	3.95	11.65
Al_2_O_3_–hBN core–shell composite granules	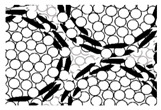	3.95	14.90

## Data Availability

Not applicable.

## Supplementary Materials

The following supporting information can be downloaded at: https://www.mdpi.com/article/10.3390/nano13010199/s1. Figure S1: SEM image of an Al_2_O_3_ core region of a Al_2_O_3_–hBN CS composites granule indicating the presence of only Al_2_O_3_ particles, Figure S2: SEM images indicating the sintering of Al_2_O_3_ particles at the (a) Al_2_O_3_ core and (b) Al_2_O_3_–hBN composite shell regions, Figure S3: XRD patterns of the raw powders used. (a) Al_2_O_3_ particles and (b) hBN sheets.
