# Supplementary material for: A Novel Controlled Fabrication of Hexagonal Boron Nitride Incorporated Composite Granules Using the Electrostatic Integrated Granulation Method
Source: Nanomaterials (Basel). 2023 Jan 2;13(1):199. doi: 10.3390/nano13010199 (PMC9824452; doi:10.3390/nano13010199)
Supplement: Supplementary file 1 [file nanomaterials-13-00199-s001.zip › nanomaterials-2097006-supplementary.pdf]

nanomaterials

Supplementary Information

A Novel Controlled Fabrication of Hexagonal Boron Nitride Incorporated Composite Granules Using the Electrostatic Integrated Granulation Method

Taisei Nakazono [1], Atsushi Yokoi [2], Wai Kian Tan [2,*], Go Kawamura [1], Atsunori Matsuda [1] and Hiroyuki Muto [1,2,*]

1 Department of Electrical and Electronics Information Engineering, Toyohashi University of Technology, Toyohashi 441-8580, Aichi, Japan
2 Institute of Liberal Arts and Sciences, Toyohashi University of Technology, Toyohashi 441-8580, Aichi, Japan
* Correspondence: tan@las.tut.ac.jp (W.K.T.); muto@ee.tut.ac.jp (H.M.)

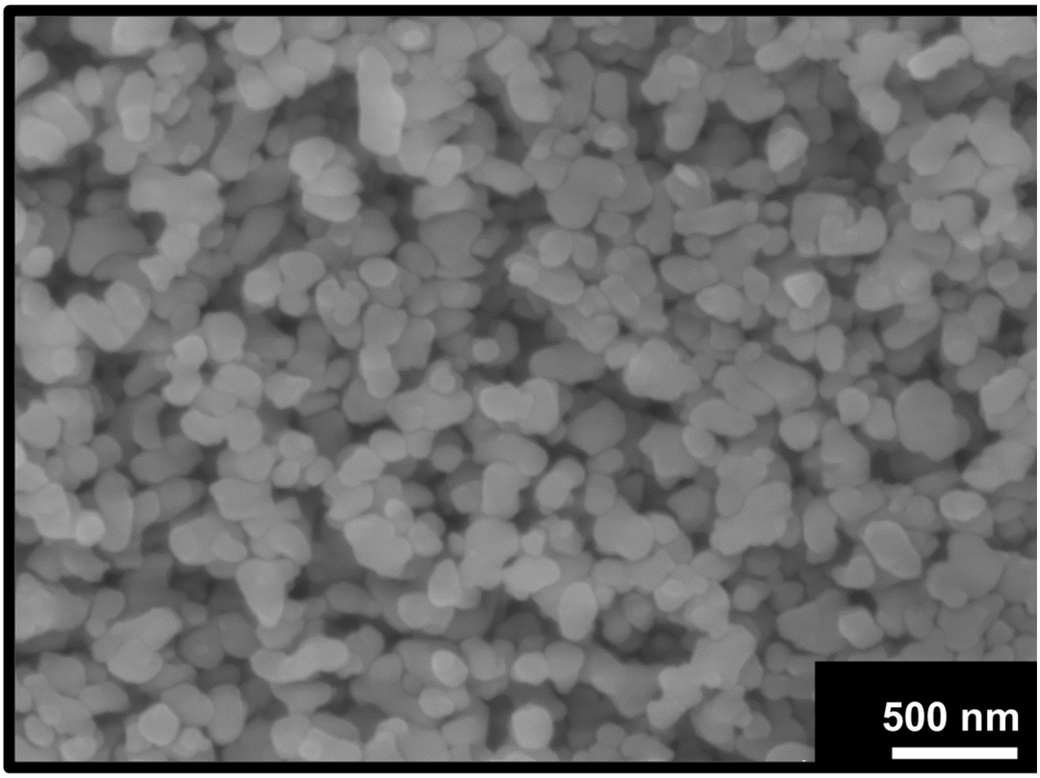

Figure S1. SEM image of an Al_2O_3 core region of a Al_2O_3–hBN CS composites granule indicating the presence of only Al_2O_3 particles.

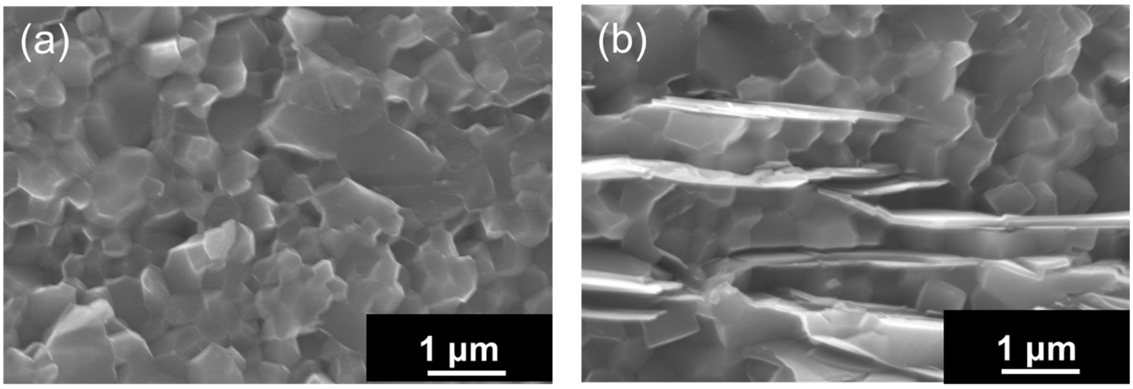

Figure S2. SEM images indicating the sintering of Al_2O_3 particles at the (a) Al_2O_3 core and (b) Al_2O_3–hBN composite shell regions.

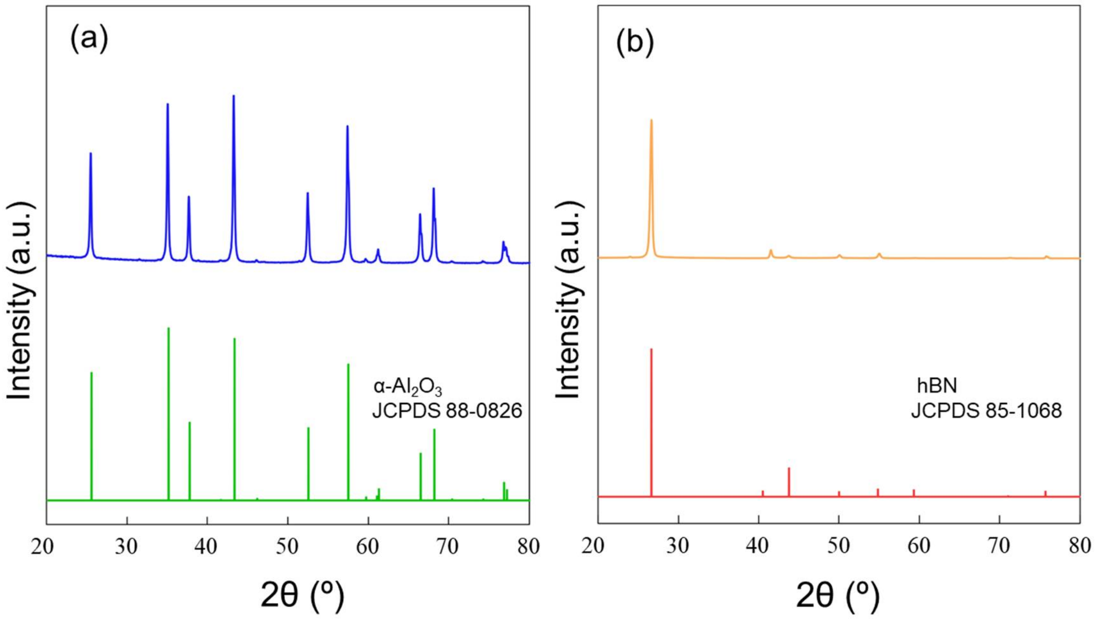

Figure S3. XRD patterns of the raw powders used. (a) Al_2O_3 particles and (b) hBN sheets.
